# Cyclin D1 expression in transitional cell carcinoma of the bladder: correlation with p53, waf1, pRb and Ki67

**DOI:** 10.1054/bjoc.2000.1557

**Published:** 2001-01

**Authors:** V M Tut, K L Braithwaite, B Angus, D E Neal, J Lunec, J K Mellon

**Affiliations:** Department of Surgery, Cancer Research Unit, Department of Pathology, University of Newcastle upon Tyne, Newcastle upon Tyne, NE2 4HH, UK

**Keywords:** cyclin D1, bladder, carcinoma, immunohistochemistry, survival

## Abstract

Normal cell proliferation is closely regulated by proteins called cyclins. One of these, cyclin D1, in combination with its corresponding cyclin-dependent kinase (cdk), is essential for G_1_/S phase transition. Cyclin/cdk complexes are generally inhibited by cyclin-dependent kinase inhibitors(ckis), some of which are induced by wild-type p53. The aims of this study were: to investigate levels of cyclin D1 expression in transitional cell carcinoma (TCC) of the bladder; to correlate these results with data concerning the expression of p53, waf1, pRb and Ki67; and to determine whether cyclin D1 expression could predict clinical outcome. Paraffin-sections from 150 newly diagnosed bladder tumours (Ta/T1 = 97; T2–T4 = 53) were stained for cyclin D1 using immunohistochemistry and a cyclin D1 index assigned. These results were correlated with data relating to the expression of p53 and waf1 by the same tumours. A representative subset of 54 tumours (Ta/T1 = 28; T2–T4 = 26) was also stained for Ki67 and 55 were stained for pRb. The clinical course of each patient was recorded and multivariate analyses of risk factors for tumour recurrence, stage progression and overall survival were performed. Positive staining for cyclin D1 was found in 83% of tumours. The staining pattern varied between tumours with nuclear, cytoplasmic or a combination of the two evident in different tumours. 89% of Ta/T1 and 74% of T2–T4 tumours showed nuclear staining with or without cytoplasmic staining. The median value for cyclin D1 staining was significantly higher in Ta/T1 tumours (41%) compared with T2–T4 tumours (8%, *P*< 0.005) with 26% of muscle-invasive tumours demonstrating absent staining. In addition, the median value for cyclin D1 staining was significantly higher in G1/G2 tumours (43%) compared with G3 tumours (14%, *P*< 0.005). There was a significant positive correlation between expression of cyclin D1 and waf1 expression (*P*< 0.0001) as well as pRb expression but not between cyclin D1 expression and expression of p53. Ki67 expression was significantly associated with increasing tumour stage (*P*< 0.005) and histological grade (*P*< 0.05) but did not correlate with cyclin D1 expression. A cyclin D1 index ≥ 8% was associated with significantly better survival in those patients with muscle-invasive disease (T2–T4). In addition, there was a significantly higher progression rate for those patients with Ta/T1 disease whose tumours demonstrated cytoplasmic cyclin D1 staining. These results indicate that cyclin D1 expression is significantly higher in low-stage, well differentiated bladder tumours and strongly correlates with waf1 expression. In a multivariate analysis, cyclin D1 expression is an independent prognostic indicator of survival in those patients with muscle-invasive disease. © 2001 Cancer Research Campaign http://www.bjcancer.com


*British Journal of Cancer* (2001) **84**(2), 270–275
© 2001 Cancer Research Campaign
doi: 10.1054/ bjoc.2000.1557, available online at http://www.idealibrary.com on IDEAL®

### Cyclin D1 expression in transitional cell carcinoma of the bladder: correlation with p53, waf1, pRb and Ki67

**VM Tut[1], KL Braithwaite[2], B Angus[3], DE Neal[1], J Lunec[2] and JK Mellon[1]**

[1]Department of Surgery; [2]Cancer Research Unit, [3]Department of Pathology, University of Newcastle upon Tyne, Newcastle upon Tyne NE2 4HH, UK

**Summary** Normal cell proliferation is closely regulated by proteins called cyclins. One of these, cyclin D1, in combination with its corresponding cyclin-dependent kinase (cdk), is essential for $G_1$/S phase transition. Cyclin/cdk complexes are generally inhibited by cyclin-dependent kinase inhibitors(ckis), some of which are induced by wild-type p53. The aims of this study were: to investigate levels of cyclin D1 expression in transitional cell carcinoma (TCC) of the bladder; to correlate these results with data concerning the expression of p53, waf1, pRb and Ki67; and to determine whether cyclin D1 expression could predict clinical outcome. Paraffin-sections from 150 newly diagnosed bladder tumours (Ta/T1 = 97; T2–T4 = 53) were stained for cyclin D1 using immunohistochemistry and a cyclin D1 index assigned. These results were correlated with data relating to the expression of p53 and waf1 by the same tumours. A representative subset of 54 tumours (Ta/T1 = 28; T2–T4 = 26) was also stained for Ki67 and 55 were stained for pRb. The clinical course of each patient was recorded and multivariate analyses of risk factors for tumour recurrence, stage progression and overall survival were performed. Positive staining for cyclin D1 was found in 83% of tumours. The staining pattern varied between tumours with nuclear, cytoplasmic or a combination of the two evident in different tumours. 89% of Ta/T1 and 74% of T2–T4 tumours showed nuclear staining with or without cytoplasmic staining. The median value for cyclin D1 staining was significantly higher in Ta/T1 tumours (41%) compared with T2–T4 tumours (8%, $P < 0.005$) with 26% of muscle-invasive tumours demonstrating absent staining. In addition, the median value for cyclin D1 staining was significantly higher in G1/G2 tumours (43%) compared with G3 tumours (14%, $P < 0.005$). There was a significant positive correlation between expression of cyclin D1 and waf1 expression ($P < 0.0001$) as well as pRb expression but not between cyclin D1 expression and expression of p53. Ki67 expression was significantly associated with increasing tumour stage ($P < 0.005$) and histological grade ($P < 0.05$) but did not correlate with cyclin D1 expression. A cyclin D1 index ≥ 8% was associated with significantly better survival in those patients with muscle-invasive disease (T2–T4). In addition, there was a significantly higher progression rate for those patients with Ta/T1 disease whose tumours demonstrated cytoplasmic cyclin D1 staining. These results indicate that cyclin D1 expression is significantly higher in low-stage, well differentiated bladder tumours and strongly correlates with waf1 expression. In a multivariate analysis, cyclin D1 expression is an independent prognostic indicator of survival in those patients with muscle-invasive disease. © 2001 Cancer Research Campaign http://www.bjcancer.com

**Keywords**: cyclin D1; bladder; carcinoma; immunohistochemistry; survival

Currently, much interest centres on the molecular processes underlying the development and progression of transitional cell carcinoma of the bladder. It is already clear that molecular pathways involving the p53 tumour-suppressor gene are of great importance in bladder cancer, especially the more aggressive types of cancer at this site (for review see Keegan et al, 1998).

Fundamental to the neoplastic process is deregulated cellular proliferation and this will almost certainly be a factor relevant to the variable biological behaviour of bladder tumours. The cyclin family of proteins which includes the mitotic cyclins, A and B, and cyclins C, D and E (associated with $G_1$/S transition) have a central role in the control of the cell cycle. They act by binding to specific catalytic subunits called cyclin-dependent kinases (cdks). One of the D cyclins, cyclin D1, in complex with cdk4 is essential for $G_1$/S phase transition and is a major positive regulator of the critical $G_1$ restriction point in the cell cycle (Sherr, 1996). The stability of this complex and translocation into the cell nucleus are regulated by the CKI, waf1/CIP1 protein (Sherr and Roberts, 1999). The cyclin D1-cdk4 complex then initiates phosphorylation of the retinoblastoma protein (pRb), disrupting its association with various E2F family members. This then allows transcription of a number of molecules necessary for DNA synthesis (Dyson, 1998). It is also at the $G_1$/S restriction point that wild-type p53, in response to cell damage, induces the transcription of the *WAF1* gene and the resulting $p21^{waf1}$ protein, a potent cdk inhibitor, binds and inactivates the cyclin/cdk complexes. This results in accumulation of the active hypophosphorylated pRb, blocking E2F-dependent transcription leading to cell cycle arrest.

Amplification of chromosome 11q13 has been found in a variety of human solid tumours including carcinoma of the bladder (Bringuier et al, 1996). Of the various genes identified at this position, the gene encoding cyclin D1 (CCND1) has been identified as the gene most consistently amplified and overexpressed (Schuuring et al, 1992). Cyclin D1 was originally isolated as the PRAD-1 oncogene (Rosenberg et al, 1991) and is known to be translocated and overexpressed in a significant subset of B-cell lymphomas.

A limited number of recent studies have begun to address the clinical significance of cyclin D1 expression in bladder cancer

*Received 21 February 2000*
*Revised 14 August 2000*
*Accepted 14 September 2000*

*Correspondence to:* JK Mellon. E-mail: j.k.mellon@ncl.ac.uk although, to date, there is not yet a consensus. Shin et al (1997), in a group of 75 tumours, reported that overexpression of cyclin D1 correlates with early recurrence in Ta/T1 tumours, while in contrast, Bringuier et al (1996) reported, in a group of 48 tumours from 46 patients, that tumours with low expression of cyclin D1 have a more aggressive clinical course.

Few studies, thus far, have examined the complete p53-waf1-cyclin D1 pathway although, in one reported series of breast cancers studied in this way, Barbareschi et al (1997) were unable to find any association between p53, cyclin D1 and the intermediary, waf1. Stein et al (1998) were, however, able to show that loss of p21 expression was an independent predictor of bladder cancer progression, while the harmful effects of abnormal p53 were abrogated by maintenance of waf1 expression. We proposed to investigate the expression of cyclin D1 in a series of human primary bladder tumours of varying stage and to relate this to clinical outcome as well as to the expression of p53, waf1, pRb and Ki67, the latter being an established marker of cell proliferation.

### PATIENTS AND METHODS

This was a retrospective study using formalin-fixed, paraffin-embedded tumour specimens obtained from 150 patients. All specimens had originally been obtained by standard endoscopic resection from patients with newly diagnosed bladder cancer between April 1986 and September 1995. Tumours were staged as defined by UICC criteria and graded according to the WHO classification. Patient demographics and tumour characteristics are summarized in Table 1. For those patients with Ta/T1 disease initially, five patients progressed to muscle-invasive disease and subsequently died (two who initially had T1G3 disease), five died of non-bladder cancer-related causes and in two the cause of death was unknown. For patients with muscle-invasive disease, 25 deaths were due to bladder cancer, two patients died of non-bladder cancer-related causes and in five the cause was unknown.

**Table 1** Patient and tumour characteristics ($n$ = 150)

| Variable | *n* | % |
|---|---|---|
| Age (years) | | |
| Mean (range) | 68 (41–91) | |
| Gender | | |
| Male | 115 | 77 |
| Female | 35 | 23 |
| Tumour grade | | |
| G1 | 20 | 13 |
| G2 | 73 | 49 |
| G3 | 57 | 38 |
| Tumour classification | | |
| pTa | 58 | 39 |
| pT1 | 39 | 26 |
| pT2 | 14 | 9 |
| pT3 | 28 | 19 |
| pT4 | 11 | 7 |
| Clinical follow-up (months) | | |
| Median (range) | 33 (1–158) | |
| Lost to follow-up | 3 | 2 |
| Ta/T1 disease with recurrence[a] | 54 | 59 |
| Time to recurrence (months) | | |
| Median (range) | 6 (3–74) | |
| Ta/T1 with progression | 9 | 6 |
| Time to progression (months) | | |
| Median (range) | 34.5 (2–158) | |
| Deaths | | |
| Ta/T1 | 12 | 8 |
| T2–T4 | 32 | 21 |
| Time to death (months) | | |
| Median (range) | 12 (0–86) | |

[a]Data concerning recurrence were not available in five patients.

### Immunohistochemistry

Immunohistochemical staining was performed on 5-µm sections of tumour using a primary monoclonal antibody directed against cyclin D1 (NCL cyclin D1-GM; Novocastra, Newcastle upon Tyne, UK). All tumours had previously been stained for p53 using NCL-DO7 monoclonal antibody (Novocastra) and waf1 using NCL-WAF1 monoclonal antibody (Novocastra). 54 tumour sections (Ta/T1 = 28, T2–T4 = 26) were also stained for Ki67 using the monoclonal antibody Ki67-mml (Novocastra) and a subset of 55 were stained for pRB using NCL-RB1 (Novocastra).

In staining for cyclin D1, sections were initially dewaxed in xylene and rehydrated in graded alcohols. Endogenous peroxidase activity was inhibited by immersing sections in $H_2O_2$ (0.3%) in methanol for 10 min. Antigen retrieval was carried out by microwaving sections for 15 min at 800 W in 0.01 citrate buffer, pH 6.0. Sections were rinsed twice in tris-buffered saline (TBS) prior to incubation with normal rabbit serum (10 min at room temperature) to inhibit non-specific binding. TBS was subsequently used to wash sections between stages. Sections were then incubated with cyclin D1 antibody (diluted 1 in 40) for 30 min at room temperature and after washing, anti-mouse IgG (diluted 1 in 500) was applied for a further 30 min. Finally, after a further washing step, streptavidin–biotinylated peroxidase complex (Dako Cambridge Ltd, UK) was applied (diluted 1 + 1 in 100). Sections were then stained with diaminobenzidine tetrahydrochloride and counterstained with haematoxylin. Sections of a mantle-cell lymphoma were used as a positive control in all staining runs and negative control sections were duplicate sections in which the primary antibody had been omitted. Five sections of normal urothelium were also stained for cyclin D1 using the same technique.

### Assessment of cyclin D1 and Ki67 staining

The staining patterns for cyclin D1 and Ki67 were assessed by two independent observers (one a histopathologist) using standard light microscopy. Nuclear and cytoplasmic staining for cyclin D1 were recorded and both results were used for the purpose of statistical analysis. For each tumour, a cyclin D1 index, defined as the percentage of cells demonstrating positive nuclear staining after counting at least 1000 tumour cells at × 400 magnification, was calculated. The intensity of staining was assessed as follows; no nuclear staining = 0, weak = 1, moderate = 2, strong = 3. Cytoplasmic staining was recorded as being present or absent. Nuclear staining alone was recorded for Ki67. For each tumour, a Ki67 index, defined as the percentage of cells demonstrating positive nuclear staining after counting at least 1000 tumour cells at × 400 magnification, was calculated.

### Assessment of p53, waf1 and pRb staining

One thousand cells in the area of highest positivity were scored using a light microscope (× 400) to obtain an average percentage positivity for p53, waf1 and pRb staining.

© 2001 Cancer Research Campaign

### Statistical analysis

The non-parametric Mann–Whitney test was used to assess the statistical significance of the relationship between the outcome of staining and tumour stage/grade. The Chi-squared test was used to assess the correlation of waf1 and cytoplasmic/nuclear cyclin D1 staining. Correlation between data sets was assessed using linear regression analysis. Survival curves were prepared using the Kaplan–Meier method with the log-rank test used to compare the difference between the curves. The multivariate analysis was performed using a forward stepwise method (Altman, 1991).

### RESULTS

### Immunohistochemical staining for cyclin D1

Sections of normal urothelium demonstrated nuclear staining in < 5% of basal cells. Nuclear staining, of variable extent, was identified in 125 of 150 (83%) sections (Figure 1). On analysing the intensity of staining, 25 specimens (17%) demonstrated absent nuclear staining, 47 (31%) had weak staining, 61 (41%) had moderate staining and 17 (11%) had strong staining. The cyclin D1 index varied widely from 0–100% (median value 38%). The median cyclin D1 index was significantly higher (41%) in Ta/T1 tumours compared with 8% for T2–T4 tumours ($P < 0.005$, Figure 2) with 26% (13 of 53) of T2–T4 tumours demonstrating absent staining. In addition, the median cyclin D1 index was significantly higher (43%) in G1/G2 tumours compared with 14% for G3 tumours ($P < 0.005$, Figure 2). Intensity of nuclear staining was significantly higher in Ta/T1 tumours compared with T2–T4 tumours ($P < 0.005$) and also significantly higher in G1/G2 tumours compared with $G_3$ tumours ($P < 0.0001$). Cytoplasmic staining of varying intensity was found either in combination with or independent of nuclear staining and was observed in 30% (15 of 53) of muscle-invasive tumours as well as 15% (15 of 97) of Ta/T1 tumours ($P = 0.60$). There was no difference in the proportion of Ta or T1 tumours with cytoplasmic cyclin D1 staining($P = 0.26$).

### Correlation between cyclin D1, p53, waf1 and pRb

Using a cut-off level of > 20%, p53 overexpression was found in 53% (28 of 53) of T2–T4 tumours and 37% (37/97) of Ta/T1 tumours. Moreover, only 5% of G1 tumours demonstrated detectable p53 staining as compared with 65% of G3 tumours. A trend towards an inverse correlation between cyclin D1 and p53 was observed although this was not statistically significant ($P = 0.053$, Table 2). There was a significant direct correlation between cyclin D1 and waf1 expression ($P < 0.0001$, Table 2). When a distinction was made in the immunolocalization of cyclin D1, we found a statistically significant correlation between nuclear waf1 staining and nuclear cyclin D1 staining ($P = 0.0011$, Table 3). pRb staining was studied in 55 cases with median values for Ta/T1 and T2–T4 tumours being 62.5% and 70% respectively. There was a

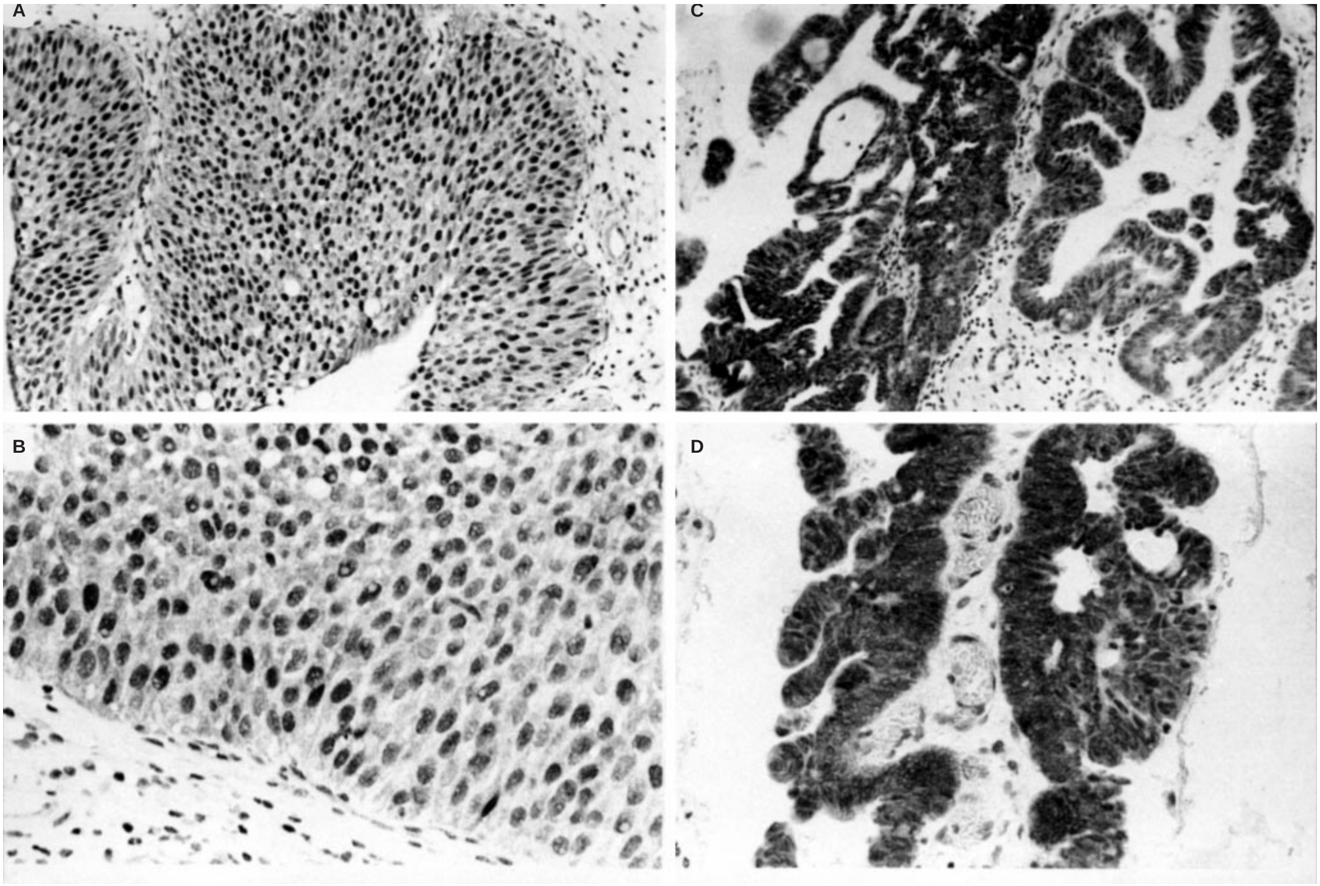

**Figure 1** (**A**) TaG1 tumour demonstrating diffuse nuclear cyclin D1 staining (× 100). (**B**) The same tumour shown at higher magnification (× 400). (**C**) Section from muscle-invasive tumour demonstrating strong cytoplasmic staining (× 100). (**D**) As **C** shown at higher magnification (× 200)

© 2001 Cancer Research Campaign

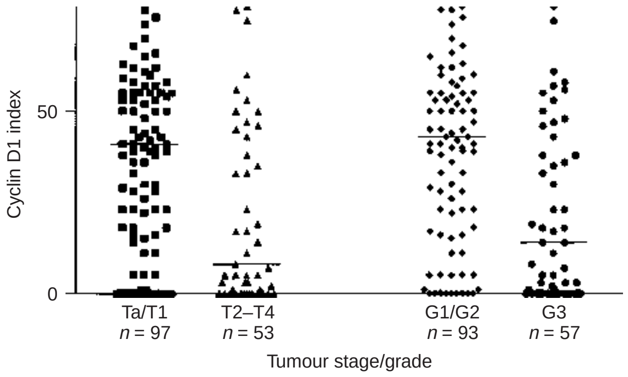

**Figure 2** Cyclin D1 expression for tumour stage/grade direct correlation between pRb and cyclin D1 in the study group as a whole ($n = 55$, $P < 0.05$, Table 2), but such a correlation was not apparent for muscle-invasive tumours when analysed separately ($P = 0.507$).

### Ki67 immunostaining

Nuclear staining for Ki67 was of variable extent with a median value of 39% positively staining tumour nuclei (range 0–95%). Only one tumour (2%) did not stain for Ki67. The median Ki67 score was significantly lower (33%) in Ta/T1 tumours compared with 51% for T2–T4 tumours ($P < 0.005$). Ki67 expression was also significantly associated with increasing tumour grade ($P < 0.05$). There was no correlation between cyclin D1 expression and Ki67 expression ($P > 0.05$, Table 2).

### Cyclin D1 and clinical outcome

Cyclin D1 expression was assessed in relation to tumour recurrence, stage progression and overall survival. No correlation was found between nuclear cyclin D1 staining and tumour recurrence or progression, however, patients with muscle-invasive disease with a cyclin D1 index of < 8% had significantly reduced survival compared with those with an index of ≥ 8% ($P = 0.011$, Figure 3). In a multivariate analysis comparing cyclin D1, p53 and tumour grade, cyclin D1 expression was found to be an independent prognostic indicator of survival ($P = 0.019$). There was no correlation between cytoplasmic staining for cyclin D1 and survival for those patients with muscle-invasive disease. In patients with Ta/T1 disease, there was no difference in recurrence rates between patients with cytoplasmic staining and those who had none. There was, however, a significant correlation between cytoplasmic staining for cyclin D1 and progression for patients with Ta/T1 disease ($P = 0.045$, Figure 4).

### DISCUSSION

In this study, we found cyclin D1 expression was significantly higher in low-stage, well differentiated bladder tumours. This finding is in agreement with Lee and colleagues who have reported overexpression of cyclin D1 in association with papillary, low-grade, non-invasive bladder tumours (Lee et al, 1997). As a consequence, one current hypothesis is that more aggressive, invasive tumours progress via a biological pathway independent of cyclin D1. To support this, Bringuier et al (1996) found that tumours with low expression of cyclin D1 were all highly aggressive. It is possible that more invasive tumours display cyclin D1 overexpression at an earlier stage in their natural history or that invasive tumours do not overexpress cyclin D1. In contrast to our study, a

**Table 2** Relationship between cyclin D1 expression and waf1, p53, pRb and Ki67 expression

| Cyclin D1 | r value | *P* value |
|---|---|---|
| Waf1 | 0.33 | 0.000011 |
| p53 | −0.19 | 0.059 |
| pRb | 0.22 | 0.031 |
| Ki67 | 0.05 | n.s. |

r = correlation coefficient; n.s. = not significant.

**Table 3** Relationship between waf1 and cyclin D1 expression (nuclear vs cytoplasmic)

| | wafl | |
|---|---|---|
| | Low < 37%[a] | High ≥ 37% |
| Cyclin D1 | | |
| Cytoplasmic | 24 | 6 |
| Nuclear | 56 | 64 |

$P = 0.0011$.
[a]37% = median waf1 score.

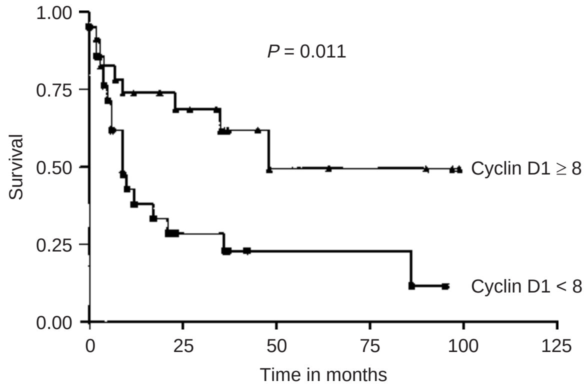

**Figure 3** Kaplan–Meier graph of survival for patients with muscle-invasive (T2–T4) disease

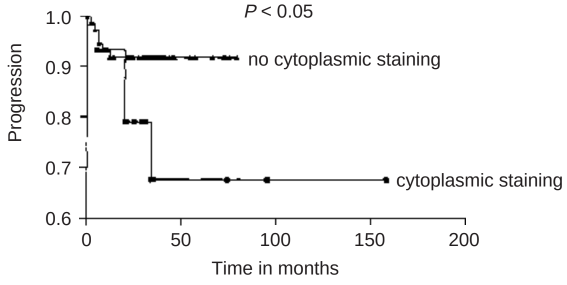

**Figure 4** Kaplan–Meier graph of progression for patients with Ta/T1 disease and cytoplasmic staining recent study of bilharzial bladder cancer reported a significant association between a cyclin D1-positive phenotype and deep muscle invasion/high tumour grade (Osman et al, 1997), although this group included squamous cell and adenocarcinomas of the bladder which may be different from the much commoner transitional cell histology.

In our series, 52% of tumours demonstrated overexpression of cyclin D1, in agreement with Shin et al (1997) who, in a smaller number of tumours, found overexpression in approximately 50% of tumours. Despite these data, the frequency of amplification of 11q13 in bladder tumours is only approximately 10–15% (Schuuring, 1995; Bringuier et al, 1996). Thus, cyclin D1 amplification is unlikely to account for the observed high rates of protein overexpression. It may be that overexpression is caused by mechanisms other than amplification, such as a mutation in the gene promoter region, post-transcriptional events or alterations of a regulatory transcription factor (Gillett et al, 1994).

An unexpected finding was the immunohistochemical localization of cyclin D1. We found cytoplasmic staining of varying intensity, either alone or in combination with nuclear staining in 30% of muscle-invasive tumours and in 15% of Ta/T1 tumours. This staining correlated with progression in those patients with Ta/T1 disease, however, we were unable to find any further relationship between this finding and clinical outcome. This finding has also been reported in a recent immunohistochemical study of cyclin D1 in transitional cell carcinoma by Suwa et al (1998). One possible explanation is that cyclin D1 or its related cdk may be inactivated by being bound to further substrates in more aggressive tumours and as a consequence loses its nuclear localization. Another possible explanation is that there may be inactivation of waf1, which normally localizes cyclin D1 complexes to the nucleus.

The significance of overexpression of cyclin D1 in relation to prognosis has now been reported in a number of tumours including adenocarcinoma of the pancreas, breast carcinoma and squamous cell carcinoma of the head and neck, with, in contrast to our study, high levels of cyclin D1 expression related to poor prognosis. In addition, Ishikawa et al (1998) most recently noted that patients with squamous cell carcinoma of the oesophagus who were cyclin D1-positive had a worse prognosis than those who were cyclin D1-negative.

Previously, in vitro studies have demonstrated that p53 induces cyclin D1, a mechanism at least partially mediated by waf1 and pRb (Chen et al, 1995). Despite this, Barbareschi et al (1997) were unable to find any association between p53, waf1 and cyclin D1 amplification/overexpression in 64 breast carcinomas. Our data are in keeping with the in vitro model, with not only a significant correlation between cyclin D1 and wafl expression, but moreover, a noticeable trend towards a negative correlation between cyclin D1 and abnormal p53. There are a number of possible reasons for the significant correlation between cyclin D1 and wafl. It has been reported that overexpression of cyclin D1 promotes cell cycle progression and differentiation, generally observed as shortened $G_1$/S transition and oncogenesis (Sherr, 1994). However, it has also been shown that cyclin D1 levels may be raised in growth-arrested cells. Pagano et al (1994) noted that an increase in cyclin D1 levels in fibroblasts led to growth arrest, and similarly Dulic et al (1993) have reported increased levels of non-functional cyclin D1 in senescent cells. Cyclin D1 may, therefore, have a dual role in growth promotion and growth arrest and in fact may reflect wild-type p53 status. Wafl may also have a dual role and the *WAF1* gene has been shown to be induced through a p53-independent mechanism following stimulation with purified growth factors or serum (Liu et al, 1996). In support of this, Zhang et al (1994) found most waf1 is located in activated cyclin/cdk complexes present in proliferating cells. More recently, it has been demonstrated that ectopic expression of cyclin D1 correlated with increased levels of waf1 which did not lead to growth arrest (Hiyama et al, 1997). In keeping with this finding, it has also been shown that, in contrast to cyclin E–cdk complexes, waf1 has a significant role in cyclin D–cdk complex assembly and translocation into the cell nucleus and also appears to stabilize the higher order complexes (Sherr and Roberts, 1999).

The retinoblastoma gene (Rb) encodes a 110 kDa nuclear protein (pRb) which is a target for phosphorylation by cyclin–cdk complexes (DeCaprio et al, 1989). Moreover, pRb has a central role in the control of cellular proliferation by regulating the $G_1$/S transition of the cell cycle (Weinberg, 1995). In early $G_1$ and $G_0$, the protein is found in its hypophosphorylated, active form. As cells progress into late $G_1$ and early S phase, pRb becomes highly phosphorylated by the cyclin–cdk complexes and remains in its phosphorylated state in the $G_2$ phase. Phosphorylation of Rb proteins inhibits their ability to bind and inactivate a family of transcription factors (E2F family) which regulate the expression of genes important for cell proliferation and DNA replication (e.g. dihydrofolate reductase, thymidylate synthase and cyclins A and E). Recently it has been suggested that the low incidence of cyclin D1 expression in high-grade bladder cancer may be due to loss of function of the Rb gene. A number of recent studies have indicated that Rb is capable of regulating cyclin D1 (Lukas et al, 1994; Muller et al, 1994). Rb is mutated or deleted in a wide array of human tumours, implying that it exerts its growth regulatory effects in a wide range of tissues. Recently, a number of groups have shown that alterations of both p53 and pRb have a cooperative negative effect in progression and survival in bladder cancer (Cote et al, 1998; Grossman et al, 1998).

In conclusion, we found that cyclin D1 expression was significantly higher in low-stage, well differentiated tumours and is an independent prognostic indicator for survival of patients with T2–T4 stage tumours. The significant association between cyclin D1 and waf1 may be a reflection of p53 status and further studies will be required to assess the functional significance of this.

### REFERENCES

Altman DG (1991) *Practical Statistics for Medical Research*. Chapman and Hall: London

Barbareschi M, Pelosio P, Caffo O, Buttitta F, Pellegrini S, Barbazza R, Dalla Palma P, Bevilacqua G and Marchetti A (1997) Cyclin-D1-Gene amplification and expression in breast carcinoma: relation with clinicopathologic characteristics and with retinoblastoma gene product, p53 and $p21^{WAF1}$ immunohistochemical expression. *Int J Cancer* **74**: 171–174

Bringuier PP, Tamimi Y, Schuuring E and Schalken J (1996) Expression of cyclin D1 and EMS1 in bladder tumours; relationship with chromosome 11q13 amplification. *Oncogene* **12**: 1747–1753

Chen X, Bargonetti J and Prives C (1995) P53, through p21 (WAF1/CIP1), induces cyclin D1 synthesis. *Cancer Res* **55**: 4257–4263

Cote RJ, Dunn MD, Chatterjee S J, Stein JP, Shi S, Tran Q, Hu SX, Xu HJ, Groshen S, Taylor CR, Skinner DG and Benedict WF (1998) Elevated and absent pRB expression is associated with bladder cancer progression and has cooperative effects with p53. *Cancer Res* **58**: 1090–1094

DeCaprio JA, Ludlow JW, Lynch D, Furukawa Y, Griffin J, Piwnica-Worms H, Huang CM and Livingston DM (1989) The product of the retinoblastoma susceptibility gene has properties of a cell cycle regulatory element. *Cell* **58**: 1085–1095

Dulic V, Drullinger LF, Lee E, Reed SI and Stein GH (1993) Altered regulation of G1 cyclins in senescent human fibroblasts: accumulation of inactive cyclin

 © 2001 Cancer Research Campaign

E-cdk2 and cyclin D1-cdk2 complexes. *Proc Natl Acad Sci USA* **90**: 11034–11038
Dyson N (1998) The regulation of E2F by pRb-family proteins. *Genes and Dev* **12**: 2245–2262
Gillett CE, Fantl V, Smith R, Fisher C, Bartek J, Dickson C, Barnes D and Peters G (1994) Amplification and over-expression of cyclin D1 in breast cancer detected by immunohistochemical staining. *Cancer Res* **54**: 1812–1817
Grossman HB, Liebert M, Antelo M, Dinney CPN, Hu S, Palmer JL and Benedict WF (1998) P53 and RB expression predict progression in T1 bladder cancer. *Clin Cancer Res* **4**: 829–834
Hiyama H, Iavarone A, Labaer J and Reeves SA (1997) Regulated ectopic expression of cyclin D1 induces transcriptional activation of the cdk inhibitor p21 gene without altering cell cycle progression. *Oncogene* **14**: 2533–2542
Ishikawa T, Furihata M, Ohtsuki Y, Murakami H, Inoue A and Ogoshi S (1998) Cyclin D1 overexpression related to retinoblastoma protein expression as a prognostic marker in human oesophageal squamous cell carcinoma. *Br J Cancer* **77**: 92–97
Keegan PE, Lunec J and Neal DE (1998) P53 and p53-regulated genes in bladder cancer. *Br J Urol* **82**: 710–720
Lee CCR, Yamamoto S, Morimura K, Wanibuchi H, Nishisaka N, Ikemoto S, Nakatani T, Wada S, Kishimoto T and Fukkushima S (1997) Significance of cyclin D1 overexpression in transitional cell carcinomas of the urinary bladder and its correlation with histopathologic features. *Cancer* **79**: 780–789
Liu Y, Martindale JL, Gorospe M and Holbrook NJ (1996) Regulation of p21 Wafl/CIP1 expression through mitogen-activated protein kinase signalling pathway. *Cancer Res* **56**: 31–35
Lukas J, Pagano M, Staskova Z, Draetta G and Bartek J (1994) Cyclin D1 protein oscillates and is essential for cell cycle progression in human tumour cell lines. *Oncogene* **9**: 707–718
Muller H, Lucas J, Schneider A, Warthoe P, Bartek J, Eilers M and Strauss M (1994) Cyclin D1 expression is regulated by the retinoblastoma protein. *Proc Natl Acad Sci USA* **91**: 2945–2949
Osman I, Scher H, Zhang Z, Soos TJ, Hamza R, Eissa S, Khaled H, Koff A and Cordon-Cardo C (1997) Expression of cyclin D1, but not cyclins E and A, is related to progression in Bilharzial bladder cancer. *Clin Cancer Res* **3**: 2247–2251
Pagano M, Theodoras AM, Tam SW and Draetta GF (1994) Cyclin D1-mediated inhibition of repair and replicative DNA synthesis in human fibroblasts. *Genes Dev* **8**: 1627–1639
Rosenberg CL, Kim HG, Shows TB, Kronenberg HM and Arnold A (1991) Rearrangement and overexpression of D11S287E, a candidate oncogene on chromosome 11q13 in benign parathyroid tumours. *Oncogene* **6**: 449–453
Schuuring E (1995) The involvement of the chromosome 11q13 region in human malignancies: cyclin D1 and EMS1 are two new candidate oncogenes – a review. *Gene* **159**: 83–96
Schuuring E, Verhoeven E, Mooi WJ and Michalides RJ (1992) Identification and cloning of two overexpressed genes, U21B31 PRAD1 and EMS1, within the amplified chromosome 11q13 region in human carcinomas. *Oncogene* **7**: 355–361
Sherr CJ (1994) G1 phase progression: cycling on cue. *Cell* **79**: 551–555
Sherr CJ (1996) Cancer cell cycles. *Science* **274**: 1672–1677
Sherr CJ, Roberts JM (1999) CDK inhibitors: positive and negative regulators of G1-phase progression. *Genes Dev* **13**: 1501–1512
Shin KY, Kong G, Kim WS, Lee TY, Woo YN and Lee JD (1997) Overexpression of cyclin D1 correlates with early recurrence in superficial bladder cancers. *Br J Cancer* **75**: 1788–1792
Stein JP, Ginsberg DA, Grossfield GD, Chatterjee SJ, Esrig D, Dickinson MG, Groshen S, Taylor CR, Jones PA, Skinner DG and Cote RJ (1998) Effect of $p21^{Waf1/Clp1}$ expression on tumour progression in bladder cancer. *J Natl Cancer Institute* **90**: 1072–1079
Suwa K, Takano Y, Iki M, Takeda M, Asakura T, Noguchi S and Masuda M (1998) Cyclin D1 protein overexpression is related to tumour differentiation, but not to tumour progression or proliferative activity, in transitional cell carcinoma of the bladder. *J Urol* **160**: 897–900
Weinberg RA (1995) The retinoblastoma protein and cell cycle control. *Cell* **81**: 323–330
Zhang H, Hannon GJ and Beach D (1994) P21-containing cyclin kinases exist in both active and inactive states. *Genes Dev* **8**: 1750–1758

© 2001 Cancer Research Campaign
